# Antimicrobial Peptide Modifications against Clinically Isolated Antibiotic-Resistant *Salmonella*

**DOI:** 10.3390/molecules26154654

**Published:** 2021-07-31

**Authors:** Suthee Mangmee, Onrapak Reamtong, Thareerat Kalambaheti, Sittiruk Roytrakul, Piengchan Sonthayanon

**Affiliations:** 1 Department of Molecular Tropical Medicine and Genetics, Faculty of Tropical Medicine, Mahidol University, Bangkok 10400, Thailand; suthee.mag@gmail.com (S.M.); onrapak.rea@mahidol.edu (O.R.); 2 Department of Microbiology and Immunology, Faculty of Tropical Medicine, Mahidol University, Bangkok 10400, Thailand; thareerat.kal@mahidol.edu; 3 Functional Ingredients and Food Innovation Research Group, National Center for Genetic Engineering and Biotechnology, Pathumthani 12120, Thailand; sittiruk@biotec.or.th; 4 Mahidol Oxford Tropical Medicine Research Unit, Faculty of Tropical Medicine, Mahidol University, Bangkok 10400, Thailand

**Keywords:** antimicrobial peptide, peptide design, structure-activity relationship, multidrug-resistant, bactericidal agent, biofilm inhibition and eradication, hemolytic activity

## Abstract

Antimicrobial peptides are promising molecules to address the global antibiotic resistance problem, however, optimization to achieve favorable potency and safety is required. Here, a peptide-template modification approach was employed to design physicochemical variants based on net charge, hydrophobicity, enantiomer, and terminal group. All variants of the scorpion venom peptide BmKn-2 with amphipathic α-helical cationic structure exhibited an increased antibacterial potency when evaluated against multidrug-resistant *Salmonella* isolates at a MIC range of 4–8 µM. They revealed antibiofilm activity in a dose-dependent manner. Sheep red blood cells were used to evaluate hemolytic and cell selectivity properties. Peptide Kn2-5R-NH_2_, dKn2-5R-NH_2_, and 2F-Kn2-5R-NH_2_ (variants with +6 charges carrying amidated C-terminus) showed stronger antibacterial activity than Kn2-5R (a variant with +5 charges bearing free-carboxyl group at C-terminus). Peptide dKn2-5R-NH_2_ (d-enantiomer) exhibited slightly weaker antibacterial activity with much less hemolytic activity (higher hemolytic concentration 50) than Kn2-5R-NH_2_ (l-enantiomer). Furthermore, peptide Kn2-5R with the least hydrophobicity had the lowest hemolytic activity and showed the highest specificity to *Salmonella* (the highest selectivity index). This study also explained the relationship of peptide physicochemical properties and bioactivities that would fulfill and accelerate progress in peptide antibiotic research and development.

## 1. Introduction

Antibiotic resistance is one of the most serious problems threatening human health worldwide [1]. Infections caused by antibiotic-resistant organisms have been rising, while the number of effective antibiotics has been declining and no new antibiotics have been discovered. Antibiotic-resistant Gram-negative bacteria, including *Salmonella*, a causative agent of food poisoning, are found to be very common human threats and the development of new therapeutic agents to combat them is in great demand [2].

Antimicrobial peptides (AMPs) have gained considerable attention for developing antibiotics to cope with this crisis. They are short amino acid chains exerting antimicrobial activity that may be derived from natural sources produced by various organisms, protein-excised fragments, and syntheses. More than 3000 AMPs are currently reported and most of them are composed of positively charged and hydrophobic amino acids [3,4]. This feature causes them to fold into an amphipathic conformation that plays a vital role in their action without memory or a specific receptor being needed when interacting with biomembranes [5]. They rapidly and simultaneously attack multiple targets, making microbes far less likely to develop resistance compared to conventional antibiotics [4,6]. 

For exerting antibacterial function, AMPs molecules generally interact with bacterial membranes through electrostatic and hydrophobic interactions [7]. They can distinguish between bacterial and mammalian cells (i.e., selectivity) on the basis of different cell membrane compositions [8]. The peptides that have high antibacterial potency and selectivity to bacteria are considered as effective, safe and suitable for clinical applications. In fact, there are quite a number of peptides that reveal weak antibacterial activity and are active against host cells causing toxicity. Therefore, peptide optimization is essential for peptide antibiotic research and development. Several studies have proven that the degrees of antibacterial potency and cell selectivity are determined by the peptides’ physicochemical properties such as length, charge, secondary structure, hydrophobicity, and amphipathicity [9]. Modifying these properties in a peptide-template to achieve preferable selective antibacterial function is a simple, straightforward, and very useful tool to redesign peptides [10,11]. However, there is no fixed or universal formula for tailoring as the activity and selectivity of AMPs to bacteria are individual-specific. 

In this study, we designed peptides using a template modification approach and tested their consequent bioactivities. Six selected AMPs that have not previously been reported for anti-*Salmonella* activity were screened for the antibacterial potential against clinical *Salmonella* isolates, and the one that showed the potential activity was used as a template. The template was modified to obtain 4 physicochemical variants resulted from positive charge increased, *C*-terminal amidated, d-enantiomerized, and hydrophobic *N*-terminal capped. Antibacterial and antibiofilm activities were evaluated using antibiotic-resistant *Salmonella*, toxicity to mammalian red blood cells was tested, selectivity index to *Salmonella* was evaluated, and the relationship between peptide physicochemical properties and bioactivities was explored.

## 2. Materials and Methods

### 2.1. Peptides

#### 2.1.1. Selection of a Peptide-Template

To search for a template, six AMPs that have never been tested against *Salmonella* were used in this study, including peptide KLK (KLKLLLLLKLK) and KLK1 (KLKLLLLLKL) derived from sapecin B of flesh fly (*Sarcophaga peregrina*) larva [12,13,14,15,16,17], BmKn-2 (FIGAIARLLSKIF), and BmKn-22 (FIGAIARLLSK) derived from scorpion (*Buthus martensii* Karsch) venom [18,19,20,21,22], and Pug-1 (LLKLFFPFLETGE) and Pug-4 (FPSFLVGR) derived from pomegranate (*Punica granatum* L.) fruit [23]. The peptides were screened for their antibacterial potential against *Salmonella* using the broth microdilution method (as detailed in Section 2.4.1). The peptide exerting the most potent anti-*Salmonella* activity was then selected as a template.

#### 2.1.2. Redesign of Peptides by Template Modifications

Peptide BmKn-2 (Kn2) was picked to be a template for further modifications. Many studies have shown that variants of individual AMPs with increased cationicity especially the one coming from an arginine (R), or stretched N-terminus with hydrophobic aromatic amino acids whether phenylalanine (F) or tryptophan (W) is correlated with enhanced antibacterial potency [24,25,26,27]. C-terminal amidation or d-amino acid incorporation into peptides to mimic natural AMPs does not only render them more potent, but also more stable [28,29]. Therefore, the template was rationally modified to produce four variants with distinct physicochemical properties. These tailorings included replacing certain positions of amino acids with R (Kn2-5R; FIRRIARLLRRIF), modifying C-terminal COOH to CONH_2_ (Kn2-5R-NH_2_; FIRRIARLLRRIF-NH_2_), replacing all L- with d-amino acids (dKn2-5R-NH_2_; firriarllrrif-NH_2_), and adding F residues at the N-terminus (2F-Kn2-5R-NH_2_; FFFIRRIARLLRRIF-NH_2_). The peptide variants were then tested for antimicrobial, hemolytic, and selectivity properties.

All peptides (>90% purity) were synthesized by and purchased from Karebay Biochem, Inc. (South Brunswick, NJ, USA). They were prepared as a stock solution (2560 µM) in a peptide solvent, 0.01% acetic acid (Sigma-Aldrich, St. Louis, MO, USA) containing 0.2% bovine serum albumin (BSA) (Sigma-Aldrich) [30], and all were stored at −20 °C. The peptides were verified their molecular weight by MALDI-TOF MS (Autoflex Speed, Bruker Daltonics, Bremen, Germany).

### 2.2. Prediction of Peptide Physicochemical Properties

The molecular weight (MW), isoelectric point (pI), and net charge (z) were calculated from the amino acids and terminal groups using the Innovagen PepCalc software (https://pepcalc.com/, accessed on 4 August 2020). The net charge (z) was considered at pH 7.0 and was the sum of positively charged amino acids (lysine: K; arginine: R), a negatively charged amino acid (glutamic acid: E), and a positive charge of amidated C-terminus (-CONH_2_). The hydrophobic ratio (HR), a percentage of hydrophobic amino acids (alanine: A; phenylalanine: F; isoleucine: I; leucine: L; valine: V), was computed using the Antimicrobial Peptide Database (APD; https://wangapd3.com/main.php, accessed on 23 November 2020). The overall hydrophobicity was predicted from the reversed-phase high-performance liquid (RP-HPLC) data by calculating a percentage of acetonitrile at where it was eluted out from the column. The consensus secondary structure was predicted based on the amino acid sequence by all available algorithms with default parameters, and the percentage of α-helix content was calculated for the peptides predicted to have an α-helix structure using PRABI-Gerland Rhone-Alpes Bioinformatic Pole Gerland Site, Institute of Biology and Protein Chemistry (https://prabi.ibcp.fr/htm/site/web/home, accessed on 4 August 2020). The helical wheel projections and hydrophobic moment (μH) depicting the amphipathicity of α-helical peptides were calculated by the heliQuest (https://heliquest.ipmc.cnrs.fr/cgi-bin/ComputParams.py, accessed on 4 August 2020).

### 2.3. Salmonella Isolates and Culture Condition

All 12 isolates of *Salmonella* used in this study were clinical isolates and anonymous left-overs from laboratory diagnoses. They were maintained in a 20% glycerol (Sigma-Aldrich) stock at −80 °C and further cultured on Mueller-Hinton agar (MHA, Oxoid, Hampshire, UK) at 37 °C for 18 h to obtain single colonies for antimicrobial assays. Their information was indicated in Table 1. This study was approved by the Ethics Committee of the Faculty of Tropical Medicine (Mahidol University, Bangkok, Thailand; MUTM-EXMPT 2017-007).

### 2.4. Antibacterial Assays

#### 2.4.1. Determination of Minimal Inhibitory Concentration (MIC)

MIC was determined using the broth microdilution method described by Hancock [30] with some modifications. Briefly, bacterial colonies grown from the MHA were prepared in MHB (Oxoid) as a 0.5 McFarland suspension (which is equal to 1 × 10^8^ CFU/mL), and was further 200-fold diluted by the MHB (5 × 10^5^ CFU/mL). The bacterial suspension (50 µL) was mixed with 2-fold serial dilutions of peptide (5.5 µL) achieving final concentrations of 256 to 1 µM using a round-bottom polypropylene (PP) 96-well plate (Greiner Bio-One). The plate was incubated at 37 °C for 18 h including incubating peptide free-bacteria as positive growth control, and MHB as negative growth control. After the incubation, the lowest concentration with no visible growth was defined as a MIC value. The assay was performed in a duplicate manner.

#### 2.4.2. Determination of Minimal Bactericidal Concentration (MBC)

The plate derived from the MIC assay was immediately tested for the MBC using a drop plate method [31]. A volume of 10 µL of the culture from individual wells at MIC, 2MIC, and 4MIC was dropped on MHA plates. The agar plates were incubated at 37 °C for 18 h, and the lowest concentration with the colony growth not over 0.1% compared to the initial concentration was indicated as a MBC value [31]. The experiment was thrice performed.

#### 2.4.3. Mode of Action

In order to determine whether the peptides have bacteriostatic or bactericidal activity, a ratio of MBC to MIC (MBC/MIC) was applied. The ratio >4 was indicative of bacteriostatic peptide, and ≤4 was inferred as bactericidal peptide [32,33].

### 2.5. Antibiofilm Assays

The effect of peptides on biofilm formation was tested according to the method described by Sandasi et al. [34] with slight modifications.

#### 2.5.1. Inhibition of Initial Cell Attachment

Bacterial suspensions of 5 × 10^5^ CFU/mL in MHB were prepared as described above. The suspensions were mixed with a peptide at final concentrations of 0.25 MBC and 0.5 MBC in a total volume of 100 µL in a round-bottom polystyrene (PS) 96-well plate (Corning, Tewksbury, MA, USA). The plates were incubated to grow biofilms for 24 h at 30 °C. The incubations of bacteria without peptide and MHB without bacteria were employed as positive (100%) control and negative (0%) control for biofilm growth, respectively. The assay was carried out twice.

#### 2.5.2. Inhibition of Preformed Biofilm

Biofilms were grown for 24 h before being treated with peptides. The preformed biofilms were achieved from incubating 100 µL of the bacterial suspension (5 × 10^5^ CFU/mL) in a round-bottom PS 96-well plate at 30 °C for 24 h. An equal volume of MHB was incubated as a negative control for biofilm formation. After the incubation, the medium was gently removed and thrice rinsed with 125 µL of 30 °C prewarmed MHB. The wells with 24 h formed biofilms were treated with 150 µL of a peptide (final concentrations; MBC and 2MBC) for 24 h at 30 °C, whereas they were instead treated with MHB as a positive control for biofilm formation. The negative biofilm control wells were incubated with new MHB. The experiment was carried out in duplicate

#### 2.5.3. Measurement of Biofilm Biomass

The biofilm biomasses obtained from the antibiofilm assay plates were quantified by the crystal violet (CV) staining method [35] with minor modifications. After incubating the plates to grow the biofilms, the growth medium and free bacterial cells were gently removed from the wells and then rinsed thrice with water. The plates were air-dried and subsequently 60 °C oven-dried for 30 min. Each well was stained with 125 µL of 0.1% CV (Merck, Burlington, MA, USA) solution for 15 min. The solution was removed from the wells and further thrice rinsed with water. The biomass bound CV was dissolved with 125 µL of 95% ethanol (Merck) for 5 min, and was transferred into a flat-bottom PS 96-well plate (Jet Biofil, Guangzhou, China). Absorbance values at 590 nm (OD_590_) of the dissolved solutions were measured by a microplate reader (Tecan Sunrise, Männedorf, Switzerland). Mean OD_590_ values were determined and all were subtracted by mean OD_590_ of MHB (negative control) prior to calculate the inhibition (%) as; ((OD_590_ (no peptide) − OD_590_ (peptide))/(OD_590_ (no peptide)) × 100.

### 2.6. Hemolytic Activity Assay

The peptide toxicity to the mammalian system was screened based on the hemolytic test by measuring the hemoglobin released from ruptured red blood cells (RBCs) [36]. In this study, sheep RBCs (sRBCs) were employed. They were thrice washed and prepared as a suspension in phosphate-buffered saline (PBS, Oxoid). A suspension of 2% sRBCs was incubated with 2-fold serial dilutions of peptide (final concentrations; 256 to 1 µM in a total volume of 200 µL) at 37 °C for 1 h. The solutions of 0.1% (final concentration) Triton X-100 (Sigma-Aldrich) and PBS containing the peptide solvent were used instead of the peptides as the controls of hemolysis at 100% and 0%, respectively. Thereafter, the post-incubated suspensions were centrifuged (1000× *g*, 3 min) to pelletize residual sRBCs. The supernatants were then transferred into a flat-bottom PS 96-well plate and were measured the absorbance at 540 nm (OD_540_) by a microplate reader. The test was conducted in triplicate and the mean OD_540_ was used to calculate the hemolysis (%) as; ((OD_540_ (peptide) − OD_540_ (PBS + peptide solvent))/(OD_540_ (0.1% Triton X-100) − OD_540_ (PBS + peptide solvent))) × 100. The hemolytic concentration 50 (HC_50_), a concentration inducing 50% hemolysis, was determined.

### 2.7. Evaluation of Selectivity Index (SI)

The SI value was evaluated to indicate the specificity or preference of the peptide action toward bacteria compared to host cells [37]. The index of each peptide was calculated by the ratio of HC_50_ to the geometric mean (GM) of MIC values obtained from multiple *Salmonella* strains tested (SI = HC_50_/GM_MIC_). A peptide with a higher SI value indicates a higher selectivity toward bacteria that reflects a higher therapeutic potential.

### 2.8. Statistical Analysis and Data Visualization

The GraphPad Prism version 7.00 (GraphPad Software, La Jolla, CA, USA) was applied for all statistical analyses and data visualizations. Replicate quantitative results were calculated and shown as mean ± SD, unless otherwise specified. The HC_50_ values were determined using nonlinear regression analysis. The paired *t*-test and repeated measures one-way ANOVA with Tukey’s multiple comparison test were applied to compare between 2 groups and among 3 or more groups, respectively. The comparisons were considered as a statistically significant difference when a *p*-value < 0.05.

## 3. Results and Discussion

In this study, the template modification approach was employed to design peptides and study the relationship between peptide physicochemical properties and bioactivities. The 6 pre-existing AMPs having no report of anti-*Salmonella* activity were screened and the most potent anti-*Salmonella* peptide was chosen as a template. The template was then modified yielding four physicochemical variants (included cationicity increased with R, C-terminal amidated, d-enantiomerized, and N-terminal stretched with hydrophobic aromatic F). The variant peptides were determined for their antimicrobial activity, toxicity to sheep red blood cells (sRBCs), and cell selectivity toward *Salmonella* compared to the sRBCs.

### 3.1. Peptides and Their Characteristics

Amino acid sequences and physicochemical properties of peptides and variants were shown in Table 2. They were all short peptides (8–15 amino acid residues) with cationicity (ranged from +1 to +6) excepting for Pug-1 which was an anionic (−1) peptide. Their hydrophobic ratio (HR) varied in a range of 50–70%. In addition for the redesigned peptides (indicated in italic letters), an order of overall hydrophobicity interpreted from the RP-HPLC data (the more percent acetonitrile (% ACN), the more hydrophobicity) was as; 2F-Kn2-5R-NH_2_ > Kn2-5R-NH_2_ > dKn2-5R-NH_2_ > Kn2-5R. Peptide KLK, KLK1, BmKn-2 (Kn2), BmKn-22, and all variants of Kn2 were predicted to form α-helix conformation with varying α-helix content (60–86.7%) whereas Pug-1 and Pug-4 were predicted to have random coil propensity.

**Figure 1:**
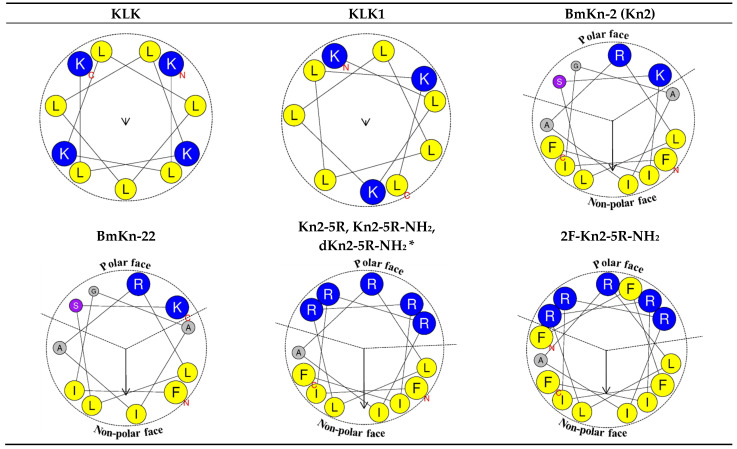
Helical wheel projections of α-helix peptides. The size of the circle is proportional to amino acid volume. Yellow indicates hydrophobic residues, blue indicates positively charged residue, purple indicates polar uncharged residue, and grey indicates the 2 smallest amino acids; glycine (G; polar uncharged residue) and alanine (A; hydrophobic residue). N and C letters represent N-terminus and C-terminus, respectively. The direction and length of the µH vector are represented by the arrow. * Opposite direction, a mirror image, to the figure presented (l-enantiomer).

Helical wheel projections of the α-helix peptides were demonstrated in Figure 1 which described amphipathicity, a property of having both a polar face and a non-polar face located on the opposite side of an α-helix [38]. The amphipathic quantity which was indicated by its hydrophobic moment (µH) [38] value ranged from 0.070 to 0.911. All α-helix peptides excepting for KLK and KLK1 exhibited 2 well-defined regions of a polar face and a non-polar face. In contrast, peptide KLK and KLK1 exhibited ambiguous polar and non-polar faces which were reflected by very small µH values. Peptide Kn2-5R, Kn2-5R-NH_2_, and dKn2-5R-NH_2_ which composed of the same amino acid sequence displayed the same helical wheel projections where dKn2-5R-NH_2_ rotated in the opposite direction (left-hand rotated) to Kn2-5R and Kn2-5R-NH_2_ (right-hand rotated). They exhibited uninterrupted polar and non-polar faces indicating perfect amphipathicity that was reflected by the highest µH value. Compared to the template (Kn-2), the variants (Kn2-5R, Kn2-5R-NH_2_, and dKn2-5R-NH_2_) depicted increased amphipathicity because the substitutions by R residues (G3R, A4R, S10R, K11R) promoted increment of the polarity of their polar face. Conversely, the 2F-Kn2-5R-NH_2_ displayed reduced amphipathicity because of disrupting polar face integrity by the hydrophobic F residue. According to all of the obtained data, these indicated that differences of changing in number and position of amino acids directly affected several peptide physicochemical properties.

### 3.2. The Peptide BmKn-2 (Kn2) Demonstrated the Most Potent Anti-Salmonella and Selected as a Template

To screen for a promising anti-*Salmonella* peptide to be used as a template, MIC values of the six selected AMPs for clinically isolated *Salmonella* were determined and used for peptide potency comparisons. Within the peptide concentration range (256–1 µM) tested, only peptide BmKn-2 (Kn2) exhibited antibacterial activity, while the other peptides did not show the activity (MIC > 256 µM) (Table S1). Kn2 was able to inhibit the visible growth of all eight *Salmonella* isolates examined, which had MIC values in a range of 64–256 µM with a geometric mean of MIC values (GM_MIC_) was 140.7 µM independently of antibiotic resistance profiles (antibiograms). This indicated that Kn2 was the only peptide active against *Salmonella*, therefore, we chose Kn2 to be a template for further modifications. This highest potency of the peptide might be a result of its having the highest α-helix content and amphipathicity. This plausible reason is consistent with other studies reporting that the antibacterial activity of amphipathic α-helical cationic AMPs increases with increased α-helicity and amphipathicity [39,40,41]. Since these conformational properties take an important part in integrating with and permeating bacterial cell membranes.

### 3.3. All Kn2 Variants Exhibited Increased Anti-Salmonella through Bactericidal Activity

Four modified variants (Kn2-5R, Kn2-5R-NH_2_, dKn2-5R-NH_2_, and 2F-Kn2-5R-NH_2_) were tested against 12 clinical isolates of *Salmonella* to evaluate their antibacterial activity (Table 3). All variants had an anti-*Salmonella* activity, with MIC values that differed by no more than twice by which it was not due to any antibiogram dependency. Peptide Kn2-5R-NH_2_ showed the highest antibacterial activity, possessing the lowest GM_MIC_ of 4 µM (Kn2-5R-NH_2_ > 2F-Kn2-5R-NH_2_ > dKn2-5R-NH_2_ > Kn2-5R). MIC values of the peptides dKn2-5R-NH_2_ and 2F-Kn2-5R-NH_2_ varied, depending on the tested isolate, from 4 to 8 µM, while the remaining variants yielded equivalent MIC values for all. In comparison with the template (Kn2), the variants exhibited 8–64 times lower MIC values, indicating their higher antibacterial potency. Furthermore, we determined MBC values of all variants by observing colony growth of the cultures at MIC, 2MIC, and 4MIC. At their MIC values, no bacterial growth was detected, which indicated that their MBC and MIC values were equal and yielded a MBC/MIC ratio of 1. This evidence denoted that all variants exerted antibacterial activity to *Salmonella* through bactericidal action at their MIC values for both antibiotic-susceptible and resistant strains including multidrug-resistant (MDR) strains. A further finding among variants was that a group with six positively charges (Kn2-5R-NH_2_, dKn2-5R-NH_2_, and 2F-Kn2-5R-NH_2_) exhibited higher potency than the peptide with five positive charges (Kn2-5R), approximately by 1.3- to 2.0-folds. From this it could be inferred that the increased anti-*Salmonella* potency is a result of an increased number of net positive charges. It is possible that an increase in the net positive charge of the peptides results in stronger binding to the negatively charged elements on the bacterial cell surface (i.e., phospholipids and lipopolysaccharides in Gram-negative bacteria), which allows more peptide molecules to accumulate on the bacterial cell membrane and be more active against the bacteria [9]. However, the charge might not a sole parameter that determines the antibacterial potency because different potencies were observed among the 6-positively charged peptides. This finding implies that parameters other than the peptide charge might also influence antibacterial potency.

In addition, the relationships between physicochemical properties and antibacterial activity of the variants were observed. We found that by replacing R residues in peptide Kn2-5R not only increased the net positive charges with decreased hydrophobicity but the replacements at those positions (G3R, A4R, S10R, K11R) which were located in the polar face of its α-helix structure also resulted in an increasing polarity of the polar face. This change consequently increased their amphipathicity, α-helicity, and anti-*Salmonella* potency. Balancing in quantity and position positively charged and hydrophobic amino acid residues appears to be a key factor to determine the conformational properties (i.e., amphipathicity and α-helicity) of amphipathic α-helical cationic AMPs. Alterations of the conformation consequently modulate their antibacterial activity [10,42,43]. Non-amino acid modification, C-terminal amidation, also modulated global properties and antibacterial function as evidenced in peptide Kn2-5R-NH_2_. It possessed an increased positive charge and overall hydrophobicity compared to its free carboxyl C-terminal variant (Kn2-5R). These factors were positively correlated with the anti-*Salmonella* activity, yielding enhanced potency. This finding is similar to observations with other amphipathic α-helical cationic AMPs, revealing that C-terminal amidation results in a greater positive charge, hydrophobicity, α-helicity, and antibacterial activity [44,45]. It displayed higher hydrophobicity and exerted more potency than its d-enantiomer variant (dKn2-5R-NH_2_). This indicates that although peptides may have the same amino acid sequence, they can have different hydrophobicities when they have different enantiomers. Also, the reduced potency of the d-enantiomer peptide might be the result of a decrease in overall hydrophobicity compared to its L-enantiomer. This evidence congruent with other studies demonstrating that d-form amino acids reduce the hydrophobicity and destabilize α-helicity [46] which could weaken the antibacterial activity. However, the effect of enantiomerization may or may not affect antimicrobial function, which depends on the individual peptides as they have unique characteristics and bioactivities [15,29]. We also noticed that adding F residues at the N-terminus of peptide 2F-Kn2-5R-NH_2_ not only increased hydrophobicity, but also yielded a decrease in amphipathicity compared to other variants. This reduced amphipathicity is caused by the disruption of the polar face of the α-helix structure by the non-polar amino acid, and resulted in its lessened anti-*Salmonella* activity. This also confirms our earlier finding that the position of amino acid residues influences the peptide conformational properties, especially the amphipathicity. Furthermore, our result is supported by previous reports that disruption of either polar or non-polar face of the α-helix structure lessens the amphipathicity and accordingly weakens the antibacterial activity of amphipathic α-helical cationic AMPs [39,47,48]. These overall findings suggest that all a peptide’s properties (i.e., charge, hydrophobicity, amphipathicity, and α-helicity) are interrelated, and modifying one of the parameters can influence the other parameters and consequently change the global properties and antibacterial activity of individual peptides.

### 3.4. All Kn2 Variants Exerted Antibiofilm Properties

**Figure 2:**
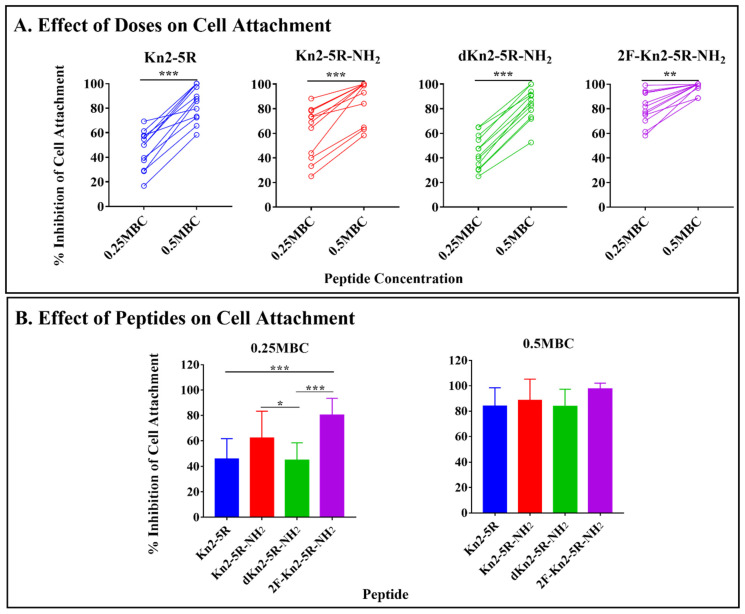

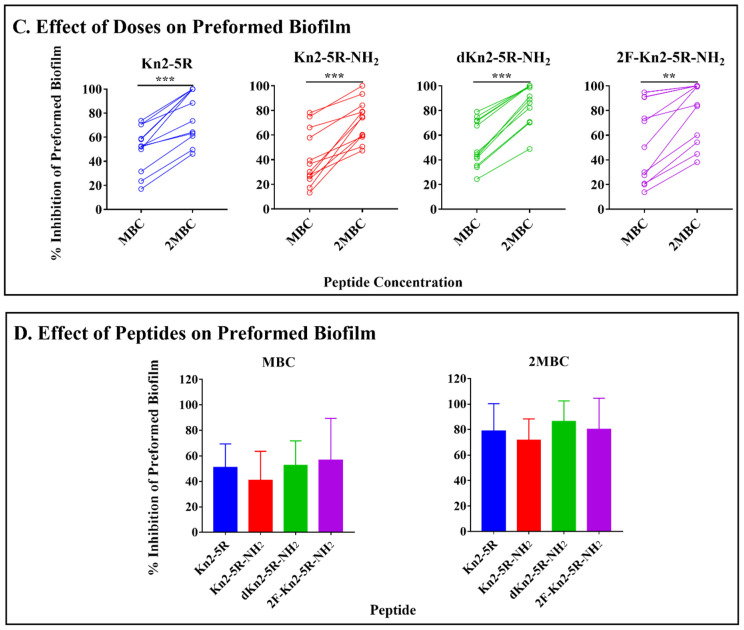
Antibiofilm activities of the modified variant peptides. Effects of peptides on biofilm biomasses of 12 clinical *Salmonella* isolates were measured by crystal violet assay. Inhibitions (%) of cell attachment treated with peptides at 0.25MBC and 0.5MBC; (**A**): effect of doses of each peptide on each isolate, (**B**): effect of each peptide at the same doses on all isolates. Inhibition (%) of 24-h preformed biofilm treated with peptides at MBC and 2MBC; (**C**): effect of doses of each peptide on each isolate, (**D**): effect of each peptide at the same doses on all strains. Paired t-test and repeated measures one-way ANOVA with Tukey’s multiple comparison test were used to compare two groups and more than two groups, respectively (* *p*-value < 0.05, ** *p*-value < 0.001, *** *p*-value < 0.0001).

To examine whether the variants have antibiofilm activity, the inhibition of initial cell attachment and 24 h-preformed biofilms of 12 clinical isolates of *Salmonella* on polystyrene surfaces were tested. We evaluated the effect of each variant at its sub-lethal concentrations (0.25 MBC and 0.5 MBC) on cell attachment, and at its lethal concentrations (MBC and 2 MBC) on the preformed biofilm by monitoring the resulting biofilm biomasses using the crystal violet assay. All variants reduced biofilm biomasses in a dose-dependent manner (Figure 2A,C; Table S2). The inhibitions (%) for both initial cell attachment and 24 h-preformed biofilm of each variant fluctuated according to the isolates treated irrespectively of antibiograms (Figure 2B,D; Table S2). According to a mean of the inhibitions (%) obtained from multiple isolates, all variants showed a reduction of cell attachment by >40% and >80% at 0.25MBC and 0.5MBC, respectively. At 0.25MBC, peptide 2F-Kn2-5R-NH_2_ exerted significantly greater inhibition of the cell attachment (80% reduction) than Kn2-5R and dKn2-5R-NH_2_ (45% reduction), while at 0.5MBC, no difference in the inhibition was observed among the variants. For the 24-h preformed biofilms, the mean inhibitions (%) of all variants at each concentration tested were not different (>40% and >70% reductions at MBC and 2MBC, respectively). These findings illustrate that the variants exerted potent antibiofilm activity towards *Salmonella* by inhibiting initial cell attachment and preformed biofilms for not only antibiotic-susceptible, but also resistant strains, including MDR strains.

As the results demonstrate that each variant with different properties (i.e., charge, hydrophobicity, amphipathicity, α-helicity) exhibited equally good antibiofilm activity, it might suggest that such properties which significantly affect antibacterial activity do not affect the antibiofilm activity. It might be possible that the fundamental factor of the activity is the result of the “FIRRIARLLRRIF” amino acid sequence common in all variants. This evidence could also imply that the mechanism of antibiofilm action might differ from the mechanism of antibacterial action despite using the same peptide. Although many mechanisms (e.g., biofilm-grown sessile cell membrane disruption, biofilm-grown EPSs degradation, inter- and intracellular signaling interference) involved in the biofilm inhibition are reported [49], however, those were independent studies and used different peptides. Therefore, it might be useful to study antibiofilm mechanisms of these variants to understand and serve as a basis for the development of preferable potent antibiofilm agents. Apart from the antibacterial and antibiofilm properties reported in this study, the motif sequence of FIRRIARLLRRIF has been reported recently in cell-penetrating peptides (CPPs) [50]. This obviously demonstrated that the peptides possess multifunctional activities which can be utilized for many applications.

### 3.5. Each Kn2 Variant Showed Different Hemolytic Activity and Cell Selectivity toward Salmonella

To primarily evaluate the mammalian cytotoxicity of the variants, their *in vitro* hemolytic activity to sRBCs was tested. The hemoglobin released from the sRBCs treated with a peptide at various concentrations (256–1 µM) for 60 min was measured. As demonstrated in Table 4; Figure S2, all variants can cause hemolysis with different toxicities and in a dose-dependent manner. For the initiation of hemolysis, peptides Kn2-5R and dKn2-5R-NH_2_ required a higher concentration (4 µM) than Kn2-5R-NH_2_ and 2F-Kn2-5R-NH_2_ (1 µM). At their MIC value, negligible hemolysis (<1%) was observed for peptide Kn2-5R and dKn2-5R-NH_2_, while it was 2% for Kn2-5R-NH_2_ and markedly high (33.7–66.7%) for 2F-Kn2-5R-NH_2_. To compare the toxicity, HC_50_ values which indicate a concentration inducing 50% hemolysis of each variant were determined. The HC_50_ value ranking in ascending order was 2F-Kn2-5R-NH_2_ < Kn2-5R-NH_2_ < dKn2-5R-NH_2_ < Kn2-5R, implying that 2F-Kn2-5R-NH_2_ had the highest toxicity (2F-Kn2-5R-NH_2_ > Kn2-5R-NH_2_ > dKn2-5R-NH_2_ > Kn2-5R).

This descending order of the toxicity was associated with a decrease in the hydrophobicity observed from the RP-HPLC data. This overall finding is in good agreement with many other studies evidencing that toxicity of AMPs to RBCs is positively correlated with their hydrophobicity whereby compounds with more hydrophobic properties exert more hemolytic activities [51,52,53]. Besides the toxicity, the cell selectivity index (SI) was determined by a ratio of HC_50_ to GM_MIC_ to evaluate the specificity of the action toward *Salmonella* versus sRBCs, reflecting the therapeutic potential. The higher the SI, the higher the antibacterial efficacy and safety. The variants demonstrated different SI values varying from 1.0 to 110.8. Three peptides (Kn2-5R, Kn2-5R-NH_2_, and dKn2-5R-NH_2_) possessed a SI value > 1 indicating their specific action against *Salmonella*. They can effectively inhibit *Salmonella* at a concentration below their HC_50_. Of these, the Kn2-5R presented the highest SI value, reflecting the most *Salmonella*-specific peptide (Kn2-5R > dKn2-5R-NH_2_ > Kn2-5R-NH_2_ > 2F-Kn2-5R-NH_2_), while 2F-Kn2-5R-NH_2_ was not specific to *Salmonella* because it exhibited equally strong antibacterial and hemolysis which yielded a SI value of 1. We found that the decrease in the SI or specificity to *Salmonella* was associated with an increase in hydrophobicity. These findings suggest that the ability to distinguish between bacterial and mammalian cells of peptides is reduced with increased hydrophobicity which is consistent with results reported for other peptides [8]. Typically, peptides can differentiate between bacterial and mammalian cells because they have different cell membrane compositions in that bacterial cells are negatively charged, while mammalian cells are neutral [8]. Therefore, the action on mammalian cells is primarily due to hydrophobic interactions and the negligible influence of electrostatic interactions. Once the peptides become more hydrophobic, they could interact more with mammalian cells and consequently cause more hemolysis through the pore-forming process. However, it should be noted that different mammalian RBCs species and cell types have different membrane compositions which might make them differently sensitive to peptides [51]. Therefore, a further comprehensive additional toxicity test on nucleated and non-nucleated cells of multiple mammalian species especially human is needed.

All results in this study that are determined under laboratory conditions might differ from physiological conditions. Therefore, further testing of these peptides under physiological conditions, especially peptide stability when exposed to salts, pH, temperatures, enzymes, and biological matrices, or even testing with other bacterial species and an animal model can be suggested. Peptide secondary structure determination using circular dichroism is suggested to elucidate the structure-activity relationship. The coexistence of many bacterial species in the same habitat is common. Therefore, testing for the activity of this peptide on mixed bacterial species needs to be further investigated to further complete the peptide study.

## 4. Conclusions

Here, we designed physicochemical variants using template modifications of a peptide Kn2 derived from scorpion venom to increase the antibacterial potency against *Salmonella* and to understand the relationship between peptide physicochemical properties and bioactivities. All of the peptides possessed an amphipathic α-helical cationic property that not only exhibited higher potent antibacterial via bactericidal action, but also demonstrated antibiofilm potential against antibiotic-resistant *Salmonella*, including MDR strains. Our study revealed the most promising peptide, Kn2-5R, that is suitable for being developed into a therapeutic agent for combating antibiotic-resistant *Salmonella* and *Salmonella* biofilm-related infections. It was the most effective and safe peptide, possessing high potency activity against *Salmonella* with low RBCs toxicity. Besides, the relationship between peptide physicochemical properties and bioactivities was described. This benefits knowledge transfer and can be used as a guide for further peptide antibiotic design and development to address the global challenge of the current antibiotic resistance crisis.

## Figures and Tables

**Table 1:** Twelve clinical *Salmonella* isolates used for antibacterial and antibiofilm assays in this study.

Isolate Code.	Month/Year	Specimen	Serogroup	Serovar	Antibiotic Resistance Profile
5	June 2002	Blood	D	Enteritidis	AMP, SXT, KZ
7	July 2002	Blood	B	Typhimurium	AMP, CN, TE, NA, SXT
11	September 2002	Blood	B	I 1,4:i:-	AMP, TE, NA, SXT, C
18	August 2002	Urine	B	Stanley	TE, SXT, C
26	June 2002	Urine	B	Typhimurium	TZP, TE, NA,
27	June 2002	Stool	B	Typhimurium	AMP, TE, NA, SXT, CIP,
55	June 2002	Blood	B	Typhimurium	AMP, SXT, C, TE
61	June 2002	Blood	D	Enteritidis	AMP, TE, NA, SXT
69	June 2002	Stool	B	Typhimurium	AMP
76	June 2002	CSF	D	Enteritidis	NA
78	July 2002	Blood	D	Enteritidis	TE, CIP, NA, SXT
107	July 2002	Stool	B	Typhimurium	Susceptible

CSF: cerebrospinal fluid. AMP: ampicillin. SXT: trimethoprim/sulfamethoxazole. KZ: cephazolin. CN: gentamicin. TE: tetracycline. NA: nalidixic acid. C: chloramphenicol. TZP: piperacillin/tazobactam. CIP: ciprofloxacin.

**Table 2:** Physicochemical properties of peptides and variants designed in this study.

Name.	Sequence	Length	Theoretical MW	Measured MW *	pI	z	HR (%)	ACN (%)	Consensus Structure	α-Helix Content (%)	µH
KLK	KLKLLLLLKLK	11	1322.81	1323.06	11.15	+4	63.0	nd	cchhhhhhhcc	63.6	0.095
KLK1	KLKLLLLLKL	10	1194.64	1194.91	10.98	+3	70.0	nd	cchhhhhhcc	60.0	0.070
BmKn-2	FIGAIARLLSKIF	13	1448.79	1449.11	11.39	+2	69.0	nd	chhhhhhhhhh?c	76.9	0.760
BmKn-22	FIGAIARLLSK	11	1188.46	1188.86	11.39	+2	63.0	nd	chhhhhhhhcc	72.7	0.699
Pug-1	LLKLFFPFLETGE	13	1553.84	1553.96	4.15	-1	53.0	nd	c??ecc??ccccc	0.0	nd
Pug-4	FPSFLVGR	8	922.08	922.66	10.59	+1	50.0	nd	cc?ee?cc	0.0	nd
*Kn2-5R*	FIRRIARLLRRIF	13	1730.16	1730.47	12.70	+5	61.0	38.9	chhhhhhhhhhhc	84.6	0.911
*Kn2-5R-NH_2_*	FIRRIARLLRRIF-NH_2_	13	1729.17	1729.46	14.00	+6	61.0	42.1	chhhhhhhhhhhc	84.6	0.911
*dKn2-5R-NH_2_*	firriarllrrif-NH_2_	13	1729.17	1729.49	14.00	+6	61.0	41.6	chhhhhhhhhhhc	84.6	0.911
*2F-Kn2-5R-NH_2_*	FFFIRRIARLLRRIF-NH_2_	15	2023.52	2023.75	14.00	+6	66.0	53.1	?hhhhhhhhhhhhhc	86.7	0.651

MW: molecular weight. pI: isoelectric point. z: net charge. HR: hydrophobic ratio. ACN: acetonitrile. µH: hydrophobic moment. nd: no data/not determined. h: α-helix. c: random coil. e: extended strand. ?: ambiguous states. Italic letters indicate redesigned peptides in this study. Lowercase amino acids indicate d-form amino acids. C-terminal amidation is presented by -NH_2_. * MW determined using MALDI-TOF MS, mass spectra shown in Figure S1.

**Table 3:** Antibacterial activity of the variant peptides shown as MIC (MBC) in µM tested against 12 clinical *Salmonella* isolates.

Isolate Code	Kn2-5R	Kn2-5R-NH_2_	dKn2-5R-NH_2_	2F-Kn2-5R-NH_2_
5	8 (8)	4 (4)	8 (8)	4 (4)
7	8 (8)	4 (4)	8 (8)	4 (4)
11	8 (8)	4 (4)	8 (8)	4 (4)
18	8 (8)	4 (4)	8 (8)	8 (8)
26	8 (8)	4 (4)	8 (8)	8 (8)
27	8 (8)	4 (4)	4 (4)	8 (8)
55	8 (8)	4 (4)	4 (4)	8 (8)
61	8 (8)	4 (4)	4 (4)	4 (4)
69	8 (8)	4 (4)	4 (4)	4 (4)
76	8 (8)	4 (4)	8 (8)	8 (8)
78	8 (8)	4 (4)	8 (8)	8 (8)
107	8 (8)	4 (4)	4 (4)	4 (4)
**GM**	8 (8)	4 (4)	5.99 (5.99)	5.66 (5.66)
**MBC/MIC ratio**	1	1	1	1

MIC: minimal inhibitory concentration. MBC: minimal bactericidal concentration. GM: geometric mean.

**Table 4:** Hemolytic activity of the variant peptides on sheep red blood cells shown in percent hemolysis and their SI toward *Salmonella*.

Peptide Concentration (µM)	Kn2-5R	Kn2-5R-NH_2_	dKn2-5R-NH_2_	2F-Kn2-5R-NH_2_
0	0	0	0	0
1	0	0.1	0	6.1
2	0	0.3	0	15
4	0.4	2 *	0.4 *	33.7 *
8	0.7 *	2.5	0.5 *	61.6 *
16	1.4	5.4	0.9	96.2
32	1.5	16.9	2.5	97.6
64	1.8	40.3	8.7	97.9
128	2.4	99.3	23.6	99.8
256	10.6	99.5	65.6	100
**HC_50_**	886.50	67.09	199.20	5.60
**SI**	110.81	16.77	33.26	0.99

HC_50_: hemolytic concentration 50. SI: selectivity index. * Hemolysis (%) at its MIC value(s).

## Data Availability

The data presented in this study are available on request from the corresponding author.

## Supplementary Materials

The following are available online. Figure S1: MALDI-TOF mass spectra of peptides and variants, Figure S2: Dose-response curve between peptide concentrations and hemolysis, Table S1: Antibacterial activity of the 6 pre-existing AMPs, Table S2: Antibiofilm activity of the variant peptides.
