# Supplementary material for: Antimicrobial Peptide Modifications against Clinically Isolated Antibiotic-Resistant Salmonella
Source: Molecules. 2021 Jul 31;26(15):4654. doi: 10.3390/molecules26154654 (PMC8348142; doi:10.3390/molecules26154654)
Supplement: Supplementary file 1 [file molecules-26-04654-s001.zip › molecules-1304840-supplementary.pdf]

Supplementary Materials

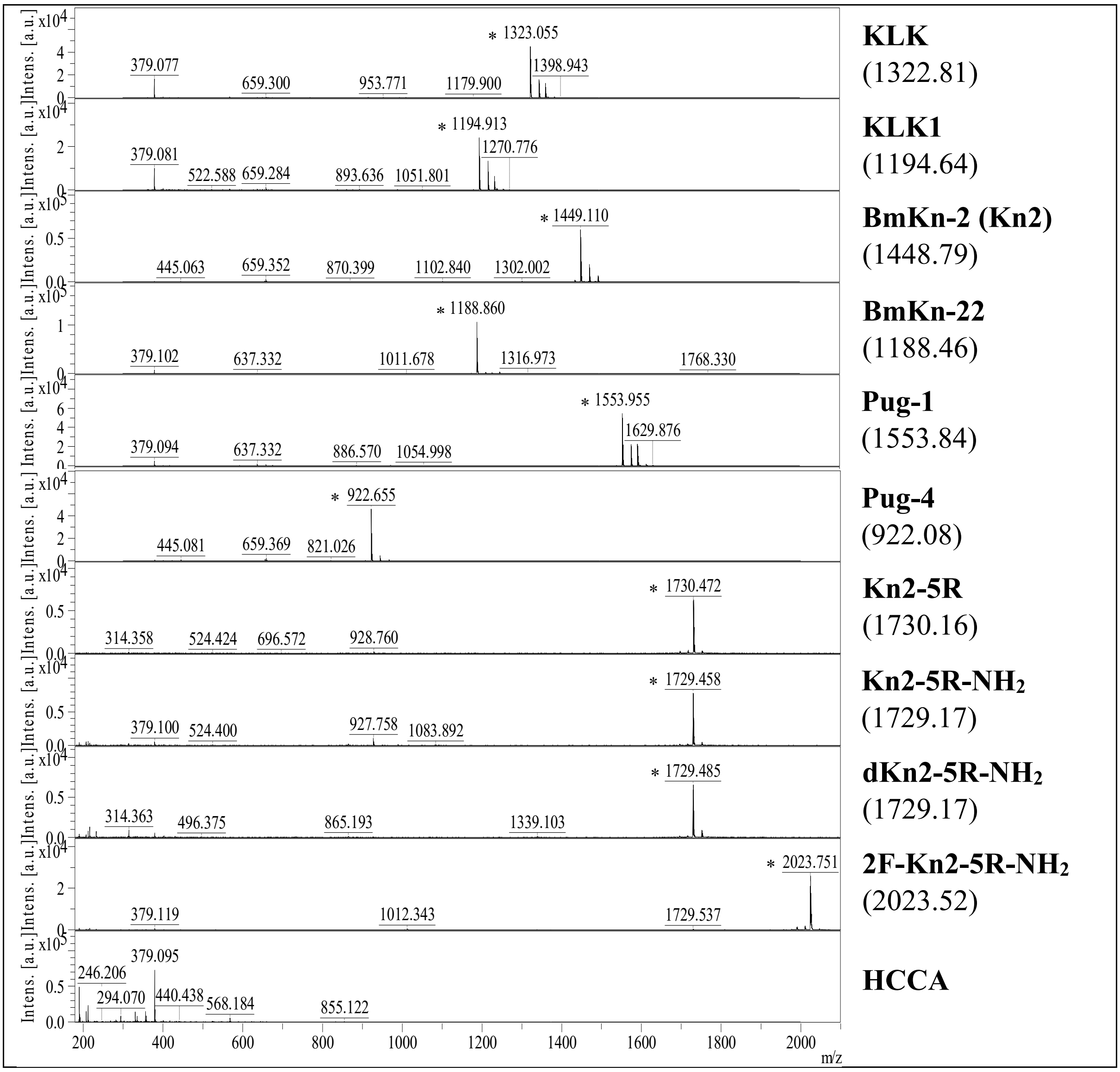

Figure S1. MALDI-TOF mass spectra of peptides and variants used in this study. The number in parentheses is its theoretical molecular weight (MW). The asterisk (*) represents MALDI-TOF measured MW (positive ion). The HCCA is a matrix used as background control.

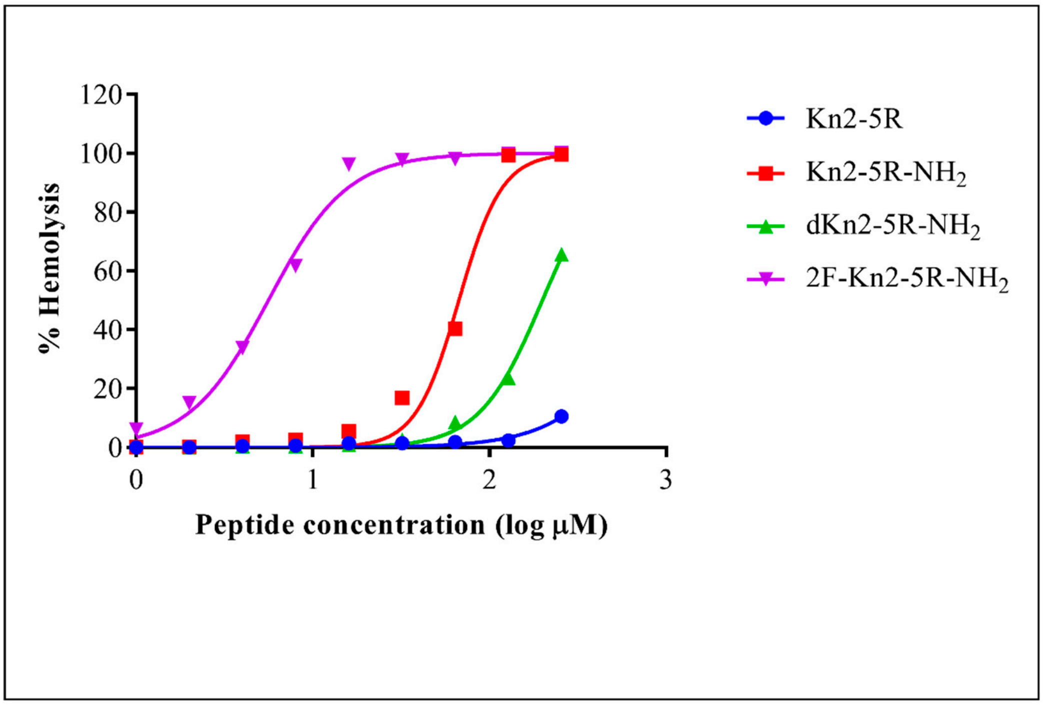

Figure S2. Dose-response curve between peptide concentrations and hemolysis (%) of the variant peptides for HC_{50} estimation.

Table S1. Antibacterial activity of the 6 pre-existing AMPs shown in µM of MIC tested with 8 clinical *Salmonella* isolates.

**Isolate Code**	**KLK**	**KLK1**	**BmKn-2**	**BmKn-22**	**Pug-1**	**Pug-4**
5	>256	>256	128	>256	>256	>256
7	>256	>256	128	>256	>256	>256
55	>256	>256	256	>256	>256	>256
61	>256	>256	256	>256	>256	>256
69	>256	>256	256	>256	>256	>256
76	>256	>256	64	>256	>256	>256
78	>256	>256	128	>256	>256	>256
107	>256	>256	64	>256	>256	>256
**GM**	>256	>256	140.7	>256	>256	>256

MIC: minimal inhibitory concentration. GM: geometric mean.

Table S2. Antibiofilm activity of the variant peptides shown in percent inhibition of cell attachment and preformed biofilm tested with 12 clinical *Salmonella* isolates.

Peptide Name	Isolate Code	Inhibition (%)			
		Cell Attachment		24-h Preformed Biofilm	
		0.5MBC	0.25MBC	2MBC	MBC
Kn2-5R	5	72.4	39.5	61.1	31.6
	7	73.2	57.9	64.3	52.2
	11	85.7	28.6	100	58.3
	18	87.5	37.5	73.6	52.7
	26	100	54.8	100	58.8
	27	58.3	16.7	100	70.6
	55	79.5	69.3	63.2	52.7
	61	89.5	57.1	100	73.8
	69	97.2	50	88.5	70.8
	76	100	50	46.2	17
	78	65.6	29.2	49.7	23.6
	107	100	61.4	100	50
Kn2-5R-NH_2	5	100	69	74.2	30.3
	7	93	73.6	79.1	26.5
	11	100	64.3	84.2	57.9
	18	58.3	25	58.6	39.5
	26	100	88.2	93.4	78.1
	27	100	79.1	100	75
	55	84.1	73.3	59.7	13.2
	61	63.2	33.3	50.6	36.7
	69	64.8	40	60.3	24.4
	76	100	79.1	78.7	66.2
	78	98.9	43.9	47.4	27.6
	107	100	78.3	75	17.3
dKn2-5R-NH_2	5	90.5	47.6	100	72
	7	94	58.3	82.1	46.2
	11	73.1	30.8	98.6	79
	18	78.9	39.5	70.2	34
	26	100	65	88.9	44.4
	27	87	34.8	86.2	41.4
	55	91.2	64.8	48.9	24.4
	61	52.5	25	91.5	42.6
	69	84.2	47.4	100	75

	76	100	54.5	100	71.4
	78	71.7	30.2	70.8	35.4
	107	82.4	41.2	100	67.7
2F-Kn2-5R-NH_2	5	88.6	75	84.6	73.7
	7	100	92.9	38.1	13.8
	11	100	77.8	60	30
	18	88.7	61.3	44.8	20.8
	26	100	93.3	100	94.7
	27	98.5	58.2	83.5	27.5
	55	100	94.4	99.4	50.3
	61	96.9	70.3	54.3	20
	69	100	81.8	100	91.1
	76	100	76.1	100	94.9
	78	100	99	100	90.9
	107	100	84.6	100	71.4

MBC: minimal bactericidal concentration.
